# Effect of insulin resistance on gonadotropin and bone mineral density in nondiabetic postmenopausal women

**DOI:** 10.3389/fendo.2023.1235102

**Published:** 2023-08-21

**Authors:** Shujin Ye, Lan Shi, Zhifen Zhang

**Affiliations:** ^1^ Department of the Fourth Clinical Medical College, Zhejiang Chinese Medical University, Hangzhou, Zhejiang, China; ^2^ Department of Obstetrics and Gynecology, The First Affiliated Hospital of Anhui Medical University, Hefei, Anhui, China; ^3^ Department of Obstetrics and Gynecology, Hangzhou Women’s Hospital (Hangzhou Maternity and Child Health Care Hospital), Hangzhou, Zhejiang, China

**Keywords:** follicle-stimulating hormone, osteoporosis, insulin resistance, postmenopausal women, insulin

## Abstract

**Objective:**

The effects of insulin resistance (IR) on bone mineral density (BMD) are unclear. This investigation aimed to assess the impact of IR and hyperinsulinemia on bone health. Determine whether IR mediates the link between follicle-stimulating hormone (FSH) and bone mass in nondiabetic postmenopausal women.

**Design:**

Retrospective cross-sectional study.

**Setting:**

Health checkup center of Hangzhou Women’s Hospital.

**Methods:**

This study comprised 437 nondiabetic postmenopausal women. BMD was evaluated using dual-energy X-rays. Fasting sera were analyzed for insulin and glucose levels, and indicators related to IR were determined. By pathway analysis, we examined the indirect effects of FSH on BMD via the mediators Homeostatic Model Assessment for insulin resistance (HOMA-IR) and fasting insulin (FINS) after correction for confounding factors.

**Result:**

After adjusting for age and body mass index (BMI) in linear regression, HOMA-IR and FINS were linked with FSH (P<0.05). IR was stronger among women in the normal BMD group than those in the osteoporosis or osteopenia group. In unadjusted models, BMD was greater in those with higher HOMA-IR and FINS (β=0.027, P=0.006 and β=0.033, P=0.003, respectively). After correcting for BMI and other possible variables, these associations remained. In addition, path models for FSH demonstrated a negative association with BMD by HOMA-IR (95% confidence interval [CI]: -0.0174 to -0.0014) and FINS (95% CI: -0.0188 to -0.002).

**Conclusion:**

Greater IR was associated with increased BMD in nondiabetic postmenopausal women, regardless of BMI and other variables. HOMA-IR or FINS could play a novel mediating role in FSH-induced BMD suppression.

## 1 Introduction

Osteoporosis is a systemic bone disease that causes increased bone fragility and fracture risk. It is characterized by a loss of bone mass and degeneration of the bone’s microarchitecture (1). Postmenopausal osteoporosis fractures and osteoporosis are prevalent, especially in older women, and hip fractures may be catastrophic (2).

One of the defining characteristics of the metabolic syndrome is peripheral IR in hepatic, skeletal muscle, and adipose tissues, which leads to hyperinsulinism. In animal models and cell line research, it has been shown that bone contains insulin receptors and that exogenous insulin activates osteoblasts and enhances indicators of bone formation, such as collagen synthesis, alkaline phosphatase, and glucose absorption (3). However, the existing research on the effects of FINS and IR on BMD was contested. Several studies have demonstrated a positive association between IR and BMD (4–6). Mendelian randomization research indicated that increased FINS and IR levels were associated with higher hip BMD (7). Some other studies have found no connection or possibly a negative association between IR and BMD (8, 9).

In postmenopausal women, the reduction in ovarian reserve causes an increase in the pituitary secretion of FSH to compensate for the decline in estradiol levels. Low FSH has been demonstrated to be linked to prediabetes and diabetes in postmenopausal women (10). IR and obesity may explain a portion of this association. Additionally, a link exists between FSH and bone mass, with tests demonstrating that a polyclonal antibody against the β-subunit of the pituitary hormone FSH enhances bone mass in mice (11). This has led to the premise that IR might play an intermediary role in the link between FSH and bone.

This study examined the association between IR, FINS, gonadotropic hormone, and BMD in postmenopausal nondiabetic women. Based on prior research, we hypothesize that as IR increases, FSH decreases and is accompanied by an increase in BMD. IR may mediate the relationship between FSH and bone. Moreover, it is vital to evaluate this link independently from the consequences of diabetes, as long-term hyperglycemia and certain anti-diabetes drugs may impair bone formation and thus have a detrimental effect (12).

## 2 Materials and methods

### 2.1 Study population

This cross-sectional study was conducted at Hangzhou Women’s Hospital between May 2016 and May 2022. Patients from the health checkup center of Hangzhou Women’s Hospital were selected using a method of purposive sampling. We eventually included 437 nondiabetic postmenopausal women with concomitant self-conscious menopausal symptoms. This research was authorized by the Hangzhou Women’s Hospital’s Ethics Review Committee.

The following were the criteria for inclusion: Women whose last menstrual period was at least 1 year ago, accompanied by conscious menopausal symptoms. Exclusion criteria included based on self-reported history of diabetes or use of diabetic medicines, or if their fasting blood glucose level ≥ 7.0 mmol/L; the use of drugs that affect bone metabolism, such as hormonal replacement agents, glucocorticoids, and thyroid or antithyroid agents; the presence of medical conditions that affect bone health, such as hyperparathyroidism, hyperprolactinemia, malignant tumor and hypercalcemia; and a history of hysterectomy and oophorectomy.

### 2.2 Biochemical assays and other measurements

After a 10-hour overnight fast, blood was obtained early in the morning. FINS, FSH, luteinizing hormone (LH), estradiol (E2), Progesterone (P), Testosterone (T), Prolactin (PRL), and thyroid stimulating hormone (TSH) were measured using the chemiluminescence method (Beckman Coulter UniCel Dxl-800). The Beckman Coulter AU5821 was used to measure fasting plasma glucose (FPG), total bilirubin (TBil), total cholesterol (TC), triglycerides (TG), high-density lipoprotein (HDL), low-density lipoprotein (LDL), lipoprotein, homocysteine (HCY), serum calcium, serum phosphorus, and C-reactive protein (CRP).

IR was computed using the following formula: HOMA-IR = [FINS (mIU/L) × Fasting Plasma Glucose (FPG, mmol/L)]/22.5; Homeostatic Model Assessment for β-cell function (HOMA-β) =20 ×FINS (mIU/L)/[FPG (mmol/L) − 3.5] (%). Triglyceride-glucose (TyG) index = Ln[FPG (mg/dl) × TG (mg/dl)]/2] (13). The metabolic score for insulin resistance (METS-IR) was calculated as follows: Ln[(2× FPG (mg/dL))+TG (mg/dL)]×BMI (kg/m2))/(Ln[HDL (mg/dL)]) (14). Impaired fasting glucose (IFG; prediabetes) was indicated by a fasting glucose level in plasma between 5.6 and 6.9 mmol/L and normal fasting glucose of < 5.6 mmol/L (15). In addition, smoking habits, habitual drinking, and physical activity were assessed according to International Osteoporosis Foundation (IOF). Smoking habits were coded as current or former smoked and never smoked. For alcohol consumption, regular drinkers were defined as those who consumed more than two units of ethanol per day. In terms of physical activity, being insufficiently active was defined as doing less than 30 minutes of physical activity per day, including housework, walking and running.

### 2.3 Bone mineral density by dual-energy X-ray absorptiometry

Dual-energy X-ray absorptiometry (DPXBravo, Ge Medical Systems China Ltd.) was utilized to measure the femoral neck BMD (g/cm^2^). The World Health Organization (WHO) definition was used to diagnose osteoporosis. Osteoporosis was defined as a T-score ≤-2.5; osteopenia as -2.5 < T-score < -1; and normal as T-score ≥-1.

### 2.4 Statistical analyses

We use the median quartile distance to characterize continuous variables and percentages to characterize categorical variables in conjunction with the Kruskal–Wallis test and the χ2 test to compare sample characteristics across the HOMA-IR index and FINS quartiles. Due to the nonnormal distributions of HOMA-IR and FINS, the ln transformation was implemented before analysis. Using multivariate linear regression, the effect of HOMA-IR and FINS on variables were analyzed after adjusting for age and BMI. The correlation between variables was analyzed by Spearman’s correlation coefficient. The Mann-Whitney U test was employed to evaluate differences between the normal BMD and osteoporosis or osteopenia groups. To determine the relationship between HOMA-IR, FINS, and BMD, we conducted a univariate regression analysis to test for linear trends and adjusted for potential covariates utilizing multiple linear regression. Age, BMI, FSH, CRP, IFG, drinking habits, smoking habits, and physical activity were all included in the final fully adjusted models. All factors were chosen because of their known or biologically reasonable links to IR and BMD (16–19).

In addition, we examined the indirect effect of FSH (X) on BMD (Y) via the mediators HOMA-IR (Mediator 1 [M1]) and FINS (M2) using path analysis [model 4 mediation, as described by Hayes]. We performed mediation analyses while controlling for age, E2, TC, and TG. The standardized indirect effect was estimated as the product of the paths from X to M and M to Y using non-parametric bootstrapping. The indirect impact was deemed statistically significant if the 95% CI did not include zero. All statistical results were analyzed by IBM-SPSS 26.0 statistics (IBM Inc., Armonk, NY, USA). P < 0.05 were considered significant.

## 3 Results

### 3.1 Cohort characteristics

The sample consisted of 437 individuals without diabetes. The characteristics of subjects by HOMA-IR and FINS quartile were shown in **Tables 1** and **2**. Women in the higher quartile had a higher BMI than women in the lower quartile (P<0.001). Moreover, the high quartile group had a worse metabolic profile than the low quartile group (lower HDL cholesterol and greater TG and FPG). FSH (P = 0.007 and P = 0.002) and LH (P = 0.008 and P = 0.009) varied substantially among the quartiles of women.

**Table 1:** Comparison of endocrine and other metabolic parameters in Postmenopausal women with different HOMA-IR levels.

Variable	HOMA-IR				
	<0.71	0.71-1.13	1.14-1.59	>1.59	P
Age (year)	53 (51–56)	54 (52–56)	54 (51–56)	53(51-56)	0.867
BMI (kg/m^2)	21.31(20.0-22.6)	21.64(20.2-22.93)	22.24(21.18-24.46)	22.85(21.34-24.69)	<0.001
FSH (IU/L)	83.03(64.15-101.3)	84.79(68.89-104.0)	73.71(58.97-90.2)	76.59(58.93-92.66)	0.007
LH (IU/L)	35.82(27.56-44.15)	35.0(26.82-44.84)	30.48(22.65-40.34)	32.36(24.53-43.19)	0.008
E2 (pg/ml)	20(20-20)	20(20-21)	20(20-25.5)	20(20-22.5)	0.513
P (ng/ml)	0.3(0.19-0.49)	0.32(0.19-0.47)	0.33(0.19-0.54)	0.29(0.18-0.43)	0.75
T (ng/ml)	0.29(0.21-0.37)	0.31(0.16-0.43)	0.31(0.21-0.41)	0.33(0.18-0.44)	0.748
PRL (ng/ml)	6.31(4.71-8.59)	6.79(4.88-8.33)	6.99(5.12-9.17)	6.18(4.81-8.52)	0.474
TBil (umol/L)	13.25(11.25-15.8)	12.4(9.7-14.2)	11.85(9.78-14.85)	11.32(9.1-13.75)	0.002
TC (mmol/L)	5.62(5.06-6.31)	5.69(4.84-6.31)	5.62(4.86-6.24)	5.82(5.16-6.77)	0.193
TG (mmol/L)	0.99(0.7-1.55)	1.1(0.83-1.55)	1.32(0.94-1.78)	1.57(1.25-2.88)	<0.001
HDL (mmol/L)	1.58(1.37-1.85)	1.55(1.33-1.71)	1.47(1.31-1.75)	1.44(1.19-1.63)	0.005
LDL (mmol/L)	2.87(2.5-3.49)	2.99(2.53-3.42)	2.99(2.5-3.57)	3.16(2.63-3.59)	0.164
lipoprotein (mg/dl)	19(10-33.5)	19.98(10-32.48)	18(9.5-36.38)	17.53(9.62-34.38)	0.934
Serum calcium (mmol/L)	2.29(2.22-2.34)	2.32(2.2-2.38)	2.31(2.21-2.37)	2.29(2.23-2.36)	0.513
Serum phosphorus (mmol/L)	1.21(1.09-1.29)	1.16(1.07-1.27)	1.2(1.12-1.28)	1.16(1.09-1.28)	0.316
HCY (umol/L)	10.25(8.2-11.7)	9.83(8.14-12.28)	9.94(8.65-12.1)	9.96(7.84-12.1)	0.948
TSH (mIU/L)	1.98(1.38-3.05)	1.72(1.17-2.79)	2.19(1.3-3.3)	1.9(1.35-3.07)	0.402
CRP (mg/L)	0.7(0.3-1.68)	0.95(0.5-1.9)	1.01(0.46-215)	1.68(0.65-2.94)	<0.001
FINS (mIU/L)	2.6(2.17-2.9)	4.1(3.6-4.42)	5.6(5.3-6.09)	9.04(7.6-10.85)	<0.001
FPG (mmol/L)	4.89(4.77-5.13)	5.1(4.83-5.44)	5.27(4.83-5.55)	5.37(5.06-5.94)	<0.001
HOMA-IR	0.57(0.47-0.64)	0.94(0.8-1.02)	1.31(1.2-1.42)	2.18(1.82-2.68)	<0.001
HOMA-β	38.04(28.2-46.09)	53.16(41.98-62.77)	64.33(51.03-81.76)	89.33(66.91-121.89)	<0.001
TyG	4.47(4.3-4.68)	4.56(4.38-4.73)	4.67(4.47-4.82)	4.8(4.65-5.04)	<0.001
METS-IR	29.5(26.5-32.0)	30.3(27.7-34.1)	32.3(29.1-36.0)	34.6(32.0-37.5)	<0.001
IFG	5/108(4.6%)	12/113(10.6%)	25/108(23.1%)	52/108(48.1%)	<0.001
FN BMD (g/cm^2^)	0.839(0.748-0.93)	0.839(0.778-0.941)	0.856(0.785-0.966)	0.882(0.794-0.963)	0.061
FN T	-0.75(-1.5-0.0)	-0.8(-1.3-0.1)	-0.6(-1.25-0.3)	-0.45(-1.1-0.3)	0.085
Physical activity	24/108(22.2%)	27/113(23.9%)	21/108(19.4%)	20/108(18.5%)	0.747
Drinking habit	3/108(2.8%)	4/113(3.5%)	2/108(1.9%)	5/108(4.6%)	0.694
Smoking habit	6/108(5.6%)	7/113(6.2%)	7/108(6.5%)	3/108(2.8%)	0.599

BMI, body mass index; FSH, follicle-stimulating hormone; LH, luteinizing hormone; E2 estradiol; P, progesterone; T, testosterone; PRL, prolactin; TBil, total bilirubin; TC, total cholesterol; TG, triglycerides; HDL, high-density lipoprotein; LDL, low-density lipoprotein; HCY, homocysteine; TSH, thyroid stimulating hormone; CRP, C-reactive protein; FINS, fasting insulin; FPG, fasting plasma glucose; HOMA-IR, Homeostatic Model Assessment for insulin resistance; HOMA-β, Homeostatic Model Assessment for β-cell function; Tyg, Triglyceride-glucose; METS-IR, the metabolic score for insulin resistance; IFG, impaired fasting glucose; FN BMD femoral neck bone mineral density.

**Table 2:** Comparison of endocrine and other metabolic parameters in Postmenopausal women with different FINS levels.

Variable	FINS				
	<3.2	3.2-4.8	4.81-6.49	>6.49	P
Age (year)	54(51-56)	54(52-56)	54(51-56)	53(51-56)	0.948
BMI (kg/m^2)	20.96(19.85-22.53)	21.7(20.2-22.81)	22.43(21.23-24.46)	22.83(21.34-24.65)	<0.001
FSH (IU/L)	85.51(65.93-102.68)	83.27(69.2-103.77)	73.15(58.3-89.2)	77.36(57.81-93.35)	0.002
LH (IU/L)	35.91(27.56-45.21)	35.44(27.23-43.76)	30.68(23.01-40.22)	32.28(24.39-43.18)	0.009
E2 (pg/ml)	20(20-20)	20(20-21)	20(20-26)	20(20-23)	0.345
P (ng/ml)	0.3(0.19-0.51)	0.32(0.2-0.46)	0.31(0.19-0.52)	0.3(0.17-0.43)	0.818
T (ng/ml)	0.29(0.19-0.36)	0.32(0.2-0.43)	0.31(0.22-0.41)	0.31(0.16-0.44)	0.406
PRL (ng/ml)	6.63(4.8-8.63)	6.5(4.81-8.04)	6.96(5.05-9.32)	6.27(4.92-8.66)	0.548
TBil (umol/L)	13.2(11.15-15.38)	12.48(9.7-14.5)	12.3(9.8-14.7)	11.3(9.1-13.59)	0.003
TC (mmol/L)	5.63(5.09-6.44)	5.72(4.89-6.45)	5.56(4.74-5.93)	5.82(5.15-6.7)	0.089
TG (mmol/L)	0.98(0.7-1.53)	1.13(0.83-1.55)	1.31(0.92-1.88)	1.55(1.25-2.98)	<0.001
HDL (mmol/L)	1.6(1.37-1.85)	1.54(1.34-1.71)	1.45(1.3-1.74)	1.43(1.19-1.63)	0.002
LDL (mmol/L)	2.96(2.51-3.5)	2.99(2.58-3.49)	2.92(2.44-3.47)	3.15(2.64-3.55)	0.196
lipoprotein (mg/dl)	20(10-33)	22(10-35)	17(10-31)	18(9-35)	0.846
Serum calcium (mmol/L)	2.28(2.21-2.34)	2.32(2.23-2.39)	2.31(2.21-2.37)	2.28(2.23-2.36)	0.195
Serum phosphorus (mmol/L)	1.21(1.1-1.28)	1.17(1.08-1.28)	1.2(1.09-1.28)	1.16(1.1-1.27)	0.630
HCY (umol/L)	10.3(8.4-11.55)	9.8(7.8-11.89)	10.2(8.9-12.3)	9.9(7.98-12.1)	0.335
TSH (mIU/L)	1.81(1.38-3.02)	1.86(1.25-2.99)	2.15(1.23-3.29)	1.92(1.42-3.02)	0.994
CRP (mg/L)	0.7(0.3-1.68)	1.05(0.43-1.82)	1.0(0.43-2.6)	1.68(0.67-2.87)	<0.001
FINS (mIU/L)	2.6(2.12-2.85)	4.04(3.5-4.4)	5.6(5.4-6.0)	9.02(7.6-10.8)	<0.001
FPG (mmol/L)	4.89(4.77-5.13)	5.1(4.83-5.44)	5.27(4.83-5.55)	5.37(5.06-5.94)	<0.001
HOMA-IR	0.57(0.46-0.63)	0.93(0.78-1.02)	1.3(1.19-1.42)	2.17(1.78-2.68)	<0.001
HOMA-β	35.67(27.76-42.05)	49.39(42.36-61.42)	65.33(53.68-81.43)	91.82(70.43-120.96)	<0.001
TyG	4.47(4.31-4.67)	4.60(4.39-4.74)	4.63(4.44-4.82)	4.77(4.64-5.04)	<0.001
METS-IR	29.4(26.3-31.8)	30.3(28.0-34.0)	32.5(29.2-36.3)	34.6(31.5-36.7)	<0.001
IFG	5/103(4.9%)	18/116(15.5%)	25/109(22.9%)	46/109(42.2%)	<0.001
FN BMD (g/cm^2^)	0.828(0.746-0.933)	0.837(0.774-0.933)	0.86(0.791-0.953)	0.892(0.794-0.971)	0.019
FN T	-0.9(-1.5 - 0)	-0.8(-1.3 - 0)	-0.6(-1.2 - 0.2)	-0.4(-1.1 - 0.3)	0.029
Physical activity	22/103(21.4%)	29/116(25%)	20/109(18.3%)	21/109(19.3%)	0.619
Drinking habit	3/103(2.9%)	4/116(3.4%)	2/109(1.8%)	5/109(4.6%)	0.71
Smoking habit	5/103(4.9%)	6/116(5.2%)	9/109(8.3%)	3/109(2.8%)	0.337

BMI, body mass index; FSH, follicle-stimulating hormone; LH, luteinizing hormone; E2 estradiol; P, progesterone; T, testosterone; PRL, prolactin; TBil, total bilirubin; TC, total cholesterol; TG, triglycerides; HDL, high-density lipoprotein; LDL, low-density lipoprotein; HCY, homocysteine; TSH, thyroid stimulating hormone; CRP, C-reactive protein; FINS, fasting insulin; FPG, fasting plasma glucose; HOMA-IR, Homeostatic Model Assessment for insulin resistance; HOMA-β, Homeostatic Model Assessment for β-cell function; Tyg, Triglyceride-glucose; METS-IR, the metabolic score for insulin resistance; IFG, impaired fasting glucose; FN BMD femoral neck bone mineral density.

### 3.2 Association of HOMA-IR, FINS with metabolic parameters

**Figure 1:**
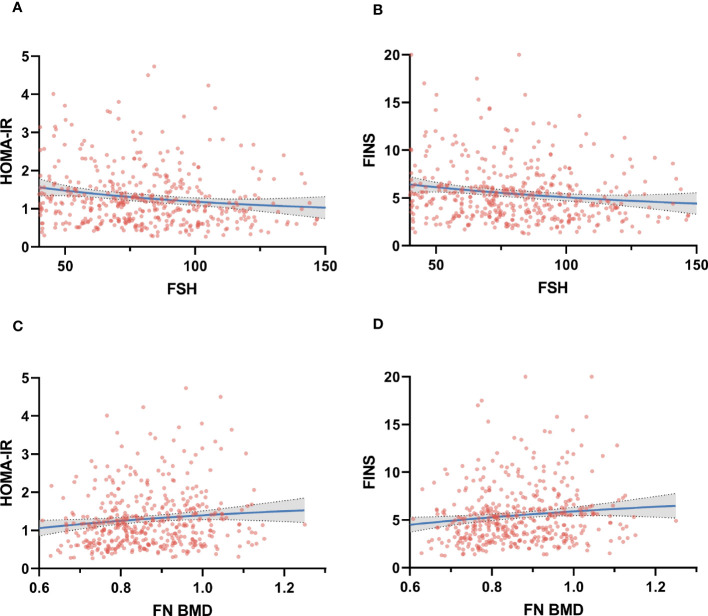
Correlation between Homeostatic Model Assessment for insulin resistance (HOMA-IR) and fasting insulin (FINS) at the follicle-stimulating hormone (FSH) (panel **A, B)** and femoral neck bone mineral density (FN BMD) (panel **C, D)**.

**Figure 2:**
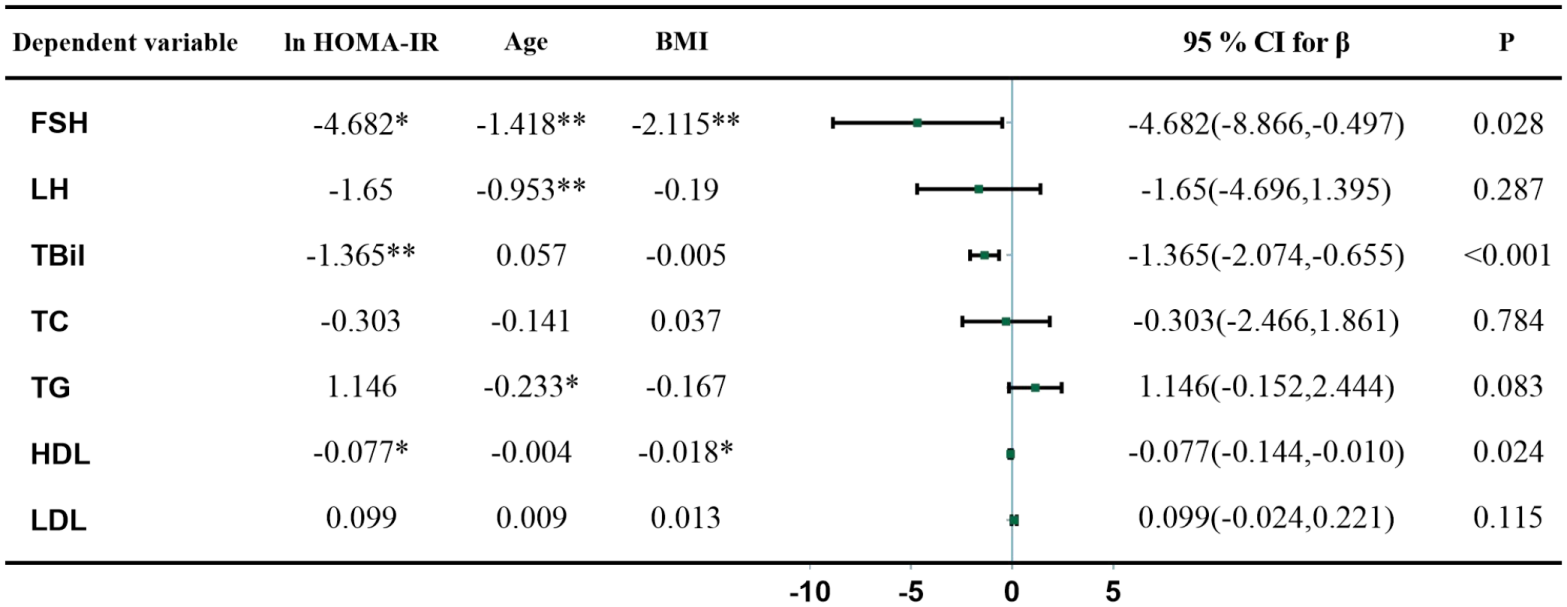
Multivariate linear regression analysis between HOMA-IR, gonadotropin, and metabolic parameters. Metabolic parameters as dependent variables, lnHOMA-IR, age, and BMI as independent variables, * and ** indicate P < 0.05 and < 0.01, respectively. β, regression coefficient; CI, confidence interval.

**Figure 3:**
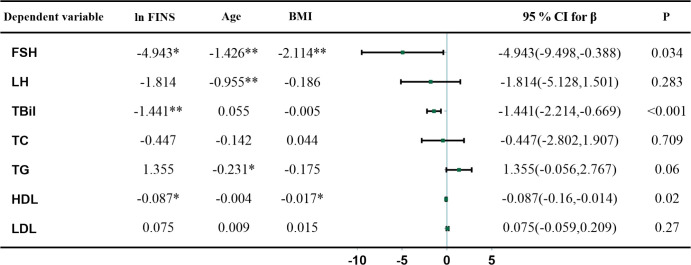
Multivariate linear regression analysis between FINS, gonadotropin, and metabolic parameters. Metabolic parameters as dependent variables, ln FINS, age, and BMI as independent variables, * and ** indicate P < 0.05 and < 0.01, respectively. β, regression coefficient; CI, confidence interval.

As FSH rose, HOMA-IR and FINS gradually declined (**Figure 1**). The results of linear regression models examining the association between HOMA-IR and metabolic parameters are summarized in **Figure 2**. After adjusting for age and BMI, higher HOMA-IR levels were associated with lower FSH (β = −4.682, P=0.028), TBil (β = −1.365, P<0.001), and HDL (β = −0.077, P=0.024). FINS also significantly negatively correlated with FSH, TBil, and HDL (**Figure 3**).

### 3.3 HOMA-IR and FINS changes in bone density

**Figure 4:**
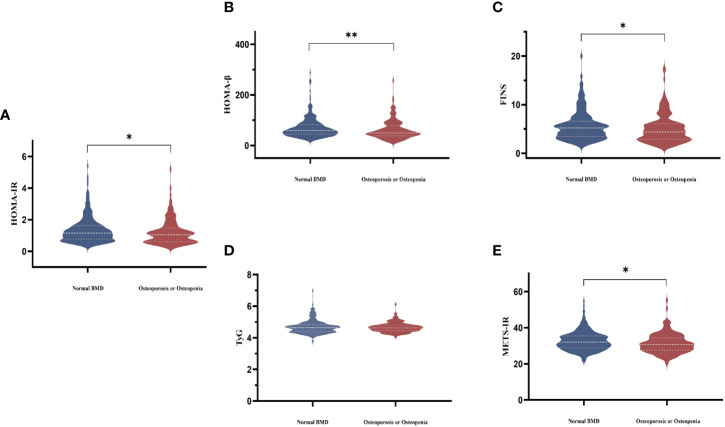
Comparisons of serum HOMA-IR **(A)**, HOMA-β **(B)**, FINS **(C)**, TyG **(D)**, and METS-IR **(E)** levels among the two groups according to BMD in postmenopausal women. The white dotted line represents the median, the lower and upper quartiles.

Femoral neck BMD was higher across increasing HOMA-IR and FINS (**Figure 1**). Femoral neck BMD and femoral neck T score significantly positively correlated with FINS, HOMA-IR, HOMA-β, METS-IR, and BMI. They were negatively correlated with FSH and LH, although the correlation was not statistical (**Table 3**). Women in the normal BMD group had greater HOMA-IR, HOMA-β, METS-IR, and FINS (P = 0.022, P =0.006, P=0.014, and P=0.011, respectively) than those in the osteoporosis or osteopenia group (**Figure 4**). The trend toward an increased TyG in those with greater BMD, although this association was insignificant (P = 0.887).

**Table 3:** Spearman’s correlation coefficients (p-value) of HOMA-IR, FINS, and other clinical and analytical variables with BMD values at the femoral neck.

variables	FN BMD	FN T score
FINS	0.151(0.002)	0.144(0.003)
FPG	-0.006(0.897)	-0.012(0.808)
HOMA-IR	0.139(0.004)	0.131(0.006)
HOMA-β	0.137(0.004)	0.134(0.005)
TyG	0.025(0.596)	0.020(0.675)
METS-IR	0.145(0.002)	0.138(0.004)
FSH	-0.071(0.14)	-0.063(0.19)
LH	-0.024(0.614)	-0.018(0.702)
HCY	0.004(0.928)	0.005(0.91)
BMI	0.201(2.2× 10^-5)	0.197(3.4× 10^-5)

**Table 4** summarized the results of the linear regression models studying the association of HOMA-IR and FINS with femoral neck BMD. Higher ln HOMA-IR levels and ln FINS levels were associated with greater BMD in model 0 (P=0.006, P=0.003). Further adjustment for age, FSH, CRP, and IFG did not attenuate the association (**Table 4**, model 1). This association was weakened after further adjustment for BMI, but statistical significance remained (**Table 4**, model 3). In fully adjusted models, the mean difference in femoral neck BMD change (95% confidence interval [CI]) per unit increase in ln HOMA-IR was 0.025 (0.003 to 0.047) g/cm^2^, and per unit increase in ln FINS was 0.027 (0.004 to 0.050) g/cm^2^. There was no evidence of collinearity when all of these variables were incorporated into multivariable models. All models’ variance inflation factors ranged between 1.008 and 1.335. To limit the potential impact of obesity, we examined the relationship between IR and BMD in individuals by conducting multivariate regression analyses with linear regression after excluding overweight and obese individuals (defined as BMI ≥23 kg/m2). In the re-adjusted model, there was a similar association between IR and BMD (**Table S1**).

**Table 4:** Independent predictors of BMD at the femoral neck as defined by multiple linear regression analyses.

variable		β Coefficient	Standard error	t	P value	95% CI for β	R2
Ln HOMA-IR	Model 0	0.027	0.010	2.762	0.006	0.008 to 0.047	0.017
	Model 1	0.030	0.011	2.696	0.007	0.008 to 0.052	0.045
	Model 2	0.031	0.011	2.760	0.006	0.009 to 0.052	0.055
	Model 3	0.025	0.011	2.235	0.026	0.003 to 0.047	0.065

Ln FINS	Model 0	0.033	0.011	3.038	0.003	0.012 to 0.054	0.021
	Model 1	0.031	0.011	2.733	0.007	0.009 to 0.054	0.045
	Model 2	0.032	0.011	2.819	0.005	0.01 to 0.055	0.056
	Model 3	0.027	0.012	2.283	0.023	0.004 to 0.050	0.066

Model 0: unadjusted.

Model 1: adjusted for age, FSH, CRP and IFG.

Model 2: additionally adjusted for physical activity, drinking habit and smoking habit.

Model 3: additionally adjusted for BMI.

### 3.4 The intermediary role of insulin resistance

**Figure 5:**
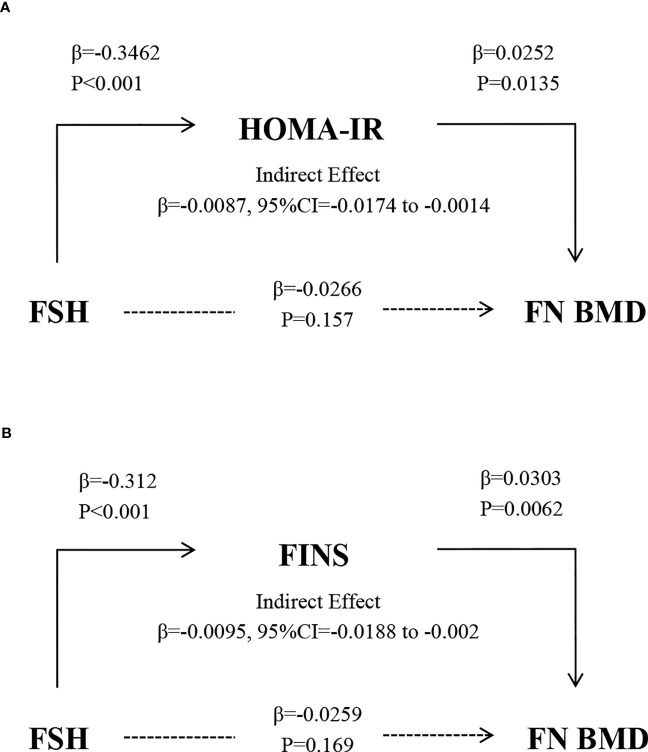
The relationship between FSH and BMD after controlling for the mediators HOMA-IR **(A)** and FINS **(B)** and adjusting for age, E2, TC, and TG. Solid and broken lines indicate statistically significant and nonsignificant connections. β, unstandardized regression coefficient.

The path models depicted in **Figure 5** illustrate the HOMA-IR and FINS-dependent relationship between FSH and femoral neck BMD, controlling for age, E2, TC, and TG. FSH was a negative predictor of HOMA-IR, and HOMA-IR was a positive predictor of femoral neck BMD (P<0.001 and P=0.0135, respectively). The indirect effect test was negative and statistically significant (β=-0.0087, 95% CI=-0.0174 to -0.0014). After adjusting for the mediator FINS, the test for indirect effects was statistically significant and negative (β = -0.0095, 95% CI = -0.0188 to -0.002).

## 4 Discussion

According to this study, higher HOMA-IR and FINS levels were substantially related to reduced FSH and increased BMD in postmenopausal nondiabetic women. The links between HOMA-IR, FINS, and BMD were maintained after controlling for potential confounders, demonstrating that IR had a protective effect on BMD within a specific range. According to our knowledge, this is the first study to investigate the role of IR as a mediator between FSH and bone. Our path analysis confirmed a negative insulin resistance-dependent connection between FSH and BMD.

In recent years, the correlation between IR and BMD has been supported by accumulating research. After adjusting for body weight and other potential covariates, Shanbhogue et al. (4) found that there was a positive correlation between HOMA-IR and total volumetric BMD, trabecular volumetric BMD, trabecular thickness, and cortical thickness at the radius and tibia, which was consistent with our findings of a higher BMD with increasing HOMA-IR. Moreover, in our investigation, the IR-related index was greater in the group with normal BMD than in the group with osteoporosis or osteopenia. Adjustment for BMI weakened the association between HOMA-IR and BMD in this study, confirming the findings of prior research (20). In addition, a longitudinal national survey of women’s health (SWAN) revealed a nonlinear relationship between mean IR levels, the rate of change in IR, and the rate of change in concurrent BMD. At lower levels of insulin resistance, a higher HOMA-IR was associated with slower BMD reduction. The population included in this study had lower HOMA-IR, and low levels of IR might promote BMD preservation (delay BMD loss), which explained the positive association between IR and BMD that we observed. Moreover, *in vitro* insulin signaling promotes osteoblast differentiation, proliferation, and function at lower insulin concentrations, supporting an anabolic effect (21). However, several researchers have reached the opposite finding, possibly due to population selection. One study included only Korean men (8), and another selected a population of women with diabetes (22), which differed from our population, and hypoglycemic medications may have harmed bone. We ultimately enrolled postmenopausal women who were not diabetic, and other factors such as age and ethnic group may also have a corresponding effect. Consequently, it can be difficult to make a reliable comparison with our research. Moreover, the relative fall in hyperinsulinemia, persistent hyperglycemia, accumulation of advanced glycation end products, and oxidative stress might contribute to the loss of bone mass and microstructure as diabetes develops (4).

Several explanations exist for the protective impact of HOMA-IR and FINS on BMD. Insulin exerts both direct and indirect effects on bone formation. On the osteoblast, insulin and insulin-like growth factor-1 (IGF-1) receptors were responsible for the direct effect (23, 24). Experiments on animals demonstrated that protein tyrosine phosphatase (Ptprv) promoted osteoprotegerin (Opg) expression via Forkhead Box O1 (FoxO1) activation by blocking insulin signaling, resulting in decreased bone resorption, which was associated with osteocalcin (Ocn) decarboxylation. Consequently, Ptprv was believed to impede glucose metabolism by reducing uncarboxylated Ocn by inhibiting bone resorption (25, 26). Several clinical studies have identified strong inverse relationships between total Ocn and IR, FPG, and obesity (27). Increased BMD in IR might be attributable to a more dramatic decrease in bone resorption than bone synthesis (28). Additionally, insulin exerted an indirect osteoprotective effect via regulating FPG levels and its effects on parathyroid hormone, IGF-1, and vitamin D (23).

We observed a significant negative correlation between FSH, HOMA-IR, and FINS (P=0.028, P=0.034). Recent investigations established that FSH was a superior biomarker for predicting the likelihood of metabolic syndrome in postmenopausal women (29, 30). Some stated that reduced FSH in obesity could be attributable to mesenchymal adipose tissue’s enhanced production of endogenous estrogens (31). Nevertheless, following adjustment for E2, lower FSH levels remained closely linked to IR, prediabetes, and diabetes (10, 32). The mechanism through which FSH affects glucose metabolism remains unclear. One possible explanation was that postmenopausal women’s elevated activin caused a progressive rise in FSH, which improved insulin sensitivity and reduced inflammation (32, 33). Liu et al. (11) observed that inhibiting the access of FSH to its receptor with an epitope-specific polyclonal antibody in mice increases bone mass (34), activation of brown adipose tissue, and enhancement of thermogenesis. The link between serum FSH, osteoporosis, and IR provides a solid foundation for our studies. Most current studies have been conducted in animal models, and further studies in humans are essential to expanding the physiological and medical roles.

Zha et al. (33) proposed that FSH-related metabolic health symptoms might negatively impact bone metabolism. We found that HOMA-IR and FINS may have a mediating effect on BMD in study participants. Meanwhile, pathway analysis showed that the indirect effect of FSH on BMD was statistically significant and negatively correlated. This might imply that FSH reduction modifies the suppression of BMD in nondiabetic postmenopausal women without diabetes through the mediating effect of elevated IR. FSH promotes bone resorption by improving the development and function of osteoclasts and promoting their survival (35). Although not statistically significant, we found a negative correlation between FSH and BMD, which may be related to the heterogeneity of our population selection and the insufficient sample size. One study showed that elevated blood FSH levels are associated with decreased BMD and bone strength and decreased lean mass, total fat mass, and visceral fat mass (17). One possible explanation was that FSH affected bone mass, adipose tissue function, energy metabolism, and cholesterol formation in both sexes via receptors with high affinity (36). These newly described metabolic and skeletal effects of FSH were consistent with our study. Therefore, we proposed that FSH modulation possibly had a therapeutic impact on various age-related diseases, including osteoporosis, obesity, and abnormal glucose metabolism.

Our use of path analysis to determine if IR was a mediator of the association between FSH and BMD in nondiabetic postmenopausal women was the primary advantage of the present investigation. In addition, unlike prior research, we utilized some of the most recent IR markers, including METS-IR and TyG, which are all positively associated with bone mass. Our study contains some limitations. The directionality of our presumed path model was based on cross-sectional data; consequently, the causal inference was inappropriate. Our lack of information on bone microarchitecture was another drawback of our research. One investigation has described the impact of obesity on bone geometry and structure using high-resolution peripheral quantitative computed tomography (37). Furthermore, the retrospective nature of the data may introduce recall bias and other biases related to data collection. Our lack of diabetic women control or comparison group limits our capacity to compare the findings with diabetic populations. Finally, we cannot be assured that our findings may be extended to other populations because the research sample consisted only of Chinese individuals.

In conclusion, elevated HOMA-IR and FINS were linked with increased BMD and decreased FSH in nondiabetic postmenopausal women. These results suggested that HOMA-IR and FINS may play a novel mediating function in inhibiting BMD by FSH, confirming previous findings. It contributed to a greater understanding of the mechanisms behind bone loss in postmenopausal women associated with FSH. Further study is required to identify measures to modulate glucose metabolism within a range to optimize metabolism and bone health in postmenopausal women.

## Data availability statement

The original contributions presented in the study are included in the article/**Supplementary Material**. Further inquiries can be directed to the corresponding author.

## Ethics statement

The studies involving human participants were reviewed and approved by The ethics review committee of Hangzhou Women’s Hospital. Written informed consent for participation was not required for this study in accordance with the national legislation and the institutional requirements.

## Author contributions

ZZ and SY contributed to the conception of the study. SY and LS were responsible for study designing, statistical analyses, and manuscript writing. ZZ contributed to revising the manuscript. SY and LS contributed to collecting data. All authors contributed to the article and approved the submitted version. SY and LS have contributed equally to this work.
