# Supplementary material for: Effect of insulin resistance on gonadotropin and bone mineral density in nondiabetic postmenopausal women
Source: Front Endocrinol (Lausanne). 2023 Aug 21;14:1235102. doi: 10.3389/fendo.2023.1235102 (PMC10475931; doi:10.3389/fendo.2023.1235102)
Supplement: Supplementary file 1 [file Table_1.docx]

| **Table S1. Independent predictors of BMD at the femoral neck as defined by multiple linear regression analyses.** | | | | | | | |
| --- | --- | --- | --- | --- | --- | --- | --- |
| variable |  | β Coefficient | Standard error | t | P value | 95 % CI for β | R2 |
| Ln HOMA-IR | Model 0 | 0.027 | 0.010 | 2.762 | 0.006 | 0.008 to 0.047 | 0.017 |
|  | Model 1 | 0.034 | 0.014 | 2.486 | 0.013 | 0.007 to 0.062 | 0.042 |
|  | Model 2 | 0.036 | 0.014 | 2.610 | 0.010 | 0.009 to 0.064 | 0.052 |
|  | Model 3 | 0.031 | 0.014 | 2.166 | 0.031 | 0.003 to 0.058 | 0.065 |
|  | | | | | | | |
| Ln FINS | Model 0 | 0.033 | 0.011 | 3.038 | 0.003 | 0.012 to 0.054 | 0.021 |
|  | Model 1 | 0.035 | 0.014 | 2.445 | 0.015 | 0.007 to 0.063 | 0.041 |
|  | Model 2 | 0.037 | 0.014 | 2.582 | 0.010 | 0.009 to 0.065 | 0.051 |
|  | Model 3 | 0.031 | 0.015 | 2.122 | 0.035 | 0.002 to 0.060 | 0.065 |

Model 0: unadjusted

Model 1: adjusted for age, FSH, CRP and IFG

Model 2: additionally adjusted for physical activity, drinking habit and smoking habit

Model 3: additionally adjusted for BMI
